# A Necessary Condition for Coexistence of Autocatalytic Replicators in a Prebiotic Environment

**DOI:** 10.3390/life3030403

**Published:** 2013-07-23

**Authors:** Andres F. Hernandez, Martha A. Grover

**Affiliations:** School of Chemical & Biomolecular Engineering, Georgia Institute of Technology, Atlanta, GA 30332, USA; E-Mail: andres.hernandez@gatech.edu

**Keywords:** chemical evolution, coexistence, diversity, replicator, prebiotic

## Abstract

A necessary, but not sufficient, mathematical condition for the coexistence of short replicating species is presented here. The mathematical condition is obtained for a prebiotic environment, simulated as a fed-batch reactor, which combines monomer recycling, variable reaction order and a fixed monomer inlet flow with two replicator types and two monomer types. An extensive exploration of the parameter space in the model validates the robustness and efficiency of the mathematical condition, with nearly 1.7% of parameter sets meeting the condition and half of those exhibiting sustained coexistence. The results show that it is possible to generate a condition of coexistence, where two replicators sustain a linear growth simultaneously for a wide variety of chemistries, under an appropriate environment. The presence of multiple monomer types is critical to sustaining the coexistence of multiple replicator types.

## 1. Introduction

Many models of early life demonstrate competition among chemical species, which leads to processes of natural selection and, eventually, to a decrease in the chemical diversity [1,2]. However, sustaining a rich and diverse chemical environment may have been necessary to develop complex biological functions. Here, an early stage in evolution is considered, prior to the emergence of any function, focused on the balance between natural selection via replication efficiency and the possibility of a sustained diversity of the chemical inventory. In this context, a space of chemical and environmental parameters is defined, where different types of autocatalytic replicators can coexist simultaneously over long periods of time.

Much of the literature on replicator dynamic modeling has focused on the case where monomer resources are lost when a replicator decomposes [1,3,4]. However, due to limited monomer resources in the environment, monomer recycling may have been important or even necessary for evolution, by reusing the monomer released after replicator decomposition [5,6,7,8]. Modern biopolymers require enzymes to catalyze their ligation and strand separation. In the absence of such enzymes, a possible prebiotic scenario is that of non-enzymatic replication of alternative chemistries with less stable backbones [9,10,11,12]. In this case, replicator degradation would be higher, so recycling would be even more important. 

The species considered in this study are short biopolymers, of order 10–100 monomers—large enough to store significant information and possess potential for catalytic function, but limited in size by the instability of their associations. Such macromolecules or assemblies are not expected to follow kinetics based on elementary reaction orders, but rather, may possess observed non-integer reaction orders dependent upon nucleation kinetics and conformation [13,14,15]. Many replicator dynamic models assume elementary reaction kinetics [3,16], in which all replicators have the same elementary reaction orders, such that only the rate constants are important for characterizing the replicator kinetics. Non-integer reaction orders on the replicator concentration have been included in a limited number of replicator dynamic models [4,17], but only for the replicator concentration, not monomer concentration. A unique feature of the model proposed here is the variable, non-integer reaction order on the monomer building materials, representing the dynamic coupling between replicator and monomer concentrations. The replicators do not interact directly with each other through cross-catalysis, only indirectly through the monomer concentrations. This coupling to the environment via the time-varying building material concentration defines a scenario in which no single replicator always has the highest replication rate. Thus, the most fit replicator is context-dependent. 

The coupling to the environment is defined via a fixed inlet flow rate of building materials into the system, different from many closed-mass models [5,8] in prebiotic chemistry. Fixed inlet flow rates have been previously considered in replicator modeling [4,16,18]. Scheuring and Szathmary [19] investigated the case where fixed flow rates are combined with a monomer recycling dynamic via the replicator decomposition. This coupling between the replicator and monomer dynamics creates a more complex scenario, which may or may not sustain the coexistence of multiple replicators. A third alternative for modeling the environmental coupling is to assume a fixed monomer composition [1]. By definition, there are no monomer dynamics in this case, simplifying the analysis. 

The combination of these three key effects—recycling, variable reaction order, and fixed monomer inlet flow—creates a dynamic scenario similar to a continuous stirred tank bioreactor with immobilized enzymes [20]. When a necessary resource is limiting, a linear growth regime is possible, in which growth is limited by the inlet flow rate of that resource [21]. For selection and evolution to be possible, coexistence should not be a transient phenomenon, but rather, the chemical and environmental conditions must sustain multiple replicators over long periods of time. This concept is used here to define coexistence as a scenario in which multiple replicators can grow with linear profiles over long periods of time. This definition of coexistence for autocatalytic replicators contrasts other biological models, like the Lotka-Volterra model, to explain mutualism [22] (*i.e.*, predator-prey model), where the explicit interaction between the species lead to a coexistence scenario with oscillatory populations. More recent work in ecological models has focused in competitive scenarios for coexistence between multiple predators and a single prey specie [23,24,25], analyzing the potential equilibria between multiple oscillatory behaviors. However, these models do not contain some of the monomer recycling features from replicator decomposition that are explored in prebiotic scenarios. 

Of course, the environment would not be expected to be constant over long periods of time, and modeling work has also considered the case of randomly fluctuating conditions [6] and direct perturbations to the replicator population [16]. In fact, random environments might promote coexistence, since each replicator type might be the most fit at a distinct sampled condition. However, in this paper, a more stringent scenario for coexistence is considered, in which the environmental conditions are constant. 

Analysis of the reaction model for the two-replicator, two-monomer case provides a criterion for coexistence, according to the linear growth definition. From the chemical parameters and the inlet conditions, one can compute whether or not the linear growth solution is feasible. A search through many parameter sets supports the case that this criterion is, in fact, necessary for coexistence. Furthermore, about half of the scenarios satisfying this condition do, in fact, exhibit coexistence in simulation. Those that do not may be rationalized in terms of the stability of the linear growth solution. This criterion for coexistence provides a design tool to guide experimental studies. It also illustrates that most of these multi-dimensional parameter sets will not lead to coexistence. However, the set of parameters enabling coexistence is still infinite and could be discovered by a complex prebiotic soup. 

## 2. Background

### 2.1. Mathematical Model of a Fed-Batch Bioreactor

**Figure 1:**
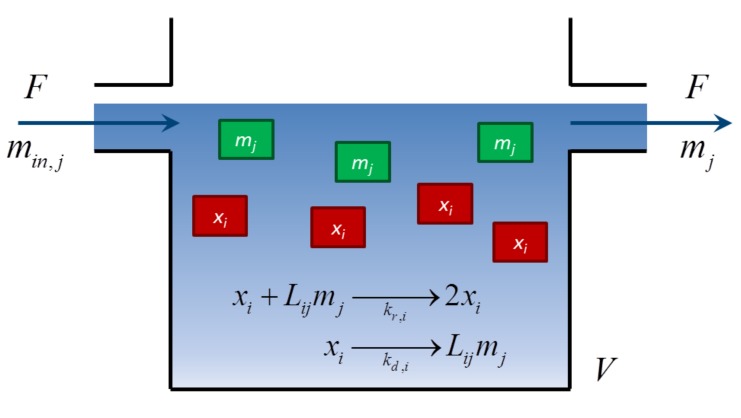
Schematic of the fed-batch system. Monomers *m_j_* flow in and out of the reactor at a volumetric flow rate, *F* . Monomers are used as building blocks by the replicators, *x_i_*, to grow in an autocatalytic process. The replicators accumulate inside the reactor volume, and they are not present in the outlet flow.

The mathematical model of a prebiotic system includes the molar concentrations of two types of chemical species: *n* replicator units, (*x_i_*, *i* = 1, ..., *n*), which grow in an autocatalytic manner, and λ monomers (*m_j_*, *j* = 1, ..., λ), which serve as building materials for the replicators. Figure 1 shows a simple schematic of the chemical system where these species coexist. The small monomer building material flows in and out of the system, at volumetric flow rate, *F* , while the larger replicating units accumulate inside the constant volume, *V* , similar to the cells in a well-mixed fed-batch bioreactor [20]. Previous studies in prebiotic chemistry argue that surface confinement and limited diffusion of the replicators may have been necessary for the replication process, in particular, for template-directed synthesis [26,27], supporting the assumption that replicators are retained in the system. The equations to describe the molar concentrations of these species are:

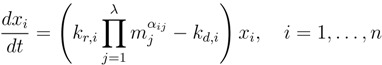
(1)

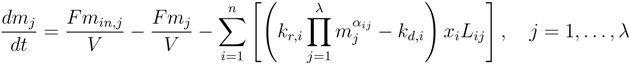
(2)


In Equations (1) and (2), the composition of the replicator units is represented by the parameters, *L_ij_*. For the purposes of this work, two different replicator types, (*x*_1_, *x*_2_, *i* = [1, 2]), and two different monomer types, (*m_a_*, *m_b_*, *j* = [*a*, *b*]), are included. Each of the replicator units has a composition of 20 building units, such that *L_ia_* + *L_ib_* = 20. Only the composition of each replicator is directly tracked in the model. The exact sequence is not directly modeled, although the impact of sequence on the kinetics could be modeled via the unique kinetic parameters for each replicator type. 

In Equation (1), the formation of a new replicator unit, *x_i_*, is represented by the constant, *k_r,i_*, whereas the dissociation of a replicator unit into free monomers is controlled by the constant, *k_d,i_*. The replicators do not interact directly, as reflected by the first-order reaction on *x_i_*, as well as the lack of *x_j_* terms in Equation (1). The autocatalytic replication rate of a replicator unit depends not only on the magnitude of *k_r,i_*, but also on its own concentration *x_i_* and the monomer concentrations, *m_j_*, inside the reactor. In simple reactions, the reaction orders are integer values associated with the stoichiometry coefficients of the corresponding reaction. This is referred to as an elementary reaction. If the mechanism of replication for replicator *R*1 is described by an elementary reaction, according to *L*_1_*_a_A* + *L*_1_*_b_B* + *R*1 → 2*R*1, then the monomer reaction orders in the reaction rate are *α*_1_*_a_* = *L*_1_*_a_* and *α*_1_*_b_* = *L*_1_*_b_*. Alternatively, Equations (1) and (2) employ an observed reaction order [20,28], where the reaction orders can be non-integer values associated with more complex reaction mechanisms than an elementary reaction. In practice, the values of *α_ij_* may be obtained from parameter fits to experimental data (an example of such mechanism is a rate-limiting step, such a nucleation of a stable core, followed by rapid growth of the final structure) that lead to such non-integer values. This type of implementation is not new to the area of prebiotic chemistry. Parabolic replicators [29,30] use a power-law reaction rate, *r* = *kx_i_*^1^^/^^2^, to represent the growth rate of replicating templates. In our work, the replication rate expression is an overall representation of the replication process without assuming a particular mechanism for how it happens. Evaluating reaction orders of the monomer concentrations in the replicator kinetics is a departure from the more common analysis of the order of the replicator concentration [4,17,29,30]. For example, a replicator could have a low sensitivity to changes in the monomer concentration of the system, and this would be modeled with a low monomer reaction order. 

Decomposition of a replicator unit, *x_i_*, limits population growth, which could be hydrolysis in a condensation reaction. This step defines the monomer recycling of the building blocks back to the available pool of monomer in the system. As shown in Equations (1) and (2), the decomposition reaction depends only on the concentration of the replicator, *x_i_*, neglecting any interaction effects with other replicators or monomers. The individual n replicators in the model are coupled via sharing of available building material, described by Equation (2). Thus, they may compete for the limited amount of monomer that is supplied to the system, or they may even cooperate to best distribute and utilize the limited building material among the various species. 

Equation (2) describes the concentration dynamics of the monomers inside the system. This equation also represents the dynamic coupling between different replicator units in the model. The monomers flow in and out of the system at a flow rate, *F* , at the same time that they are consumed and released from the replication/dissociation process of the n replicator units. One of the unique features of this model for origins of life chemistry is that one can explicitly consider environmental dynamics in prebiotic conditions (*i.e.*, starvation periods or random fluctuations of monomers in the system) using the inlet flow rate, *F* , and inlet monomer concentrations, *m_in,j_*. Changing the values of these parameters during a simulation allows one to modify the net growth rate of each of the species inside the reactor,and to explore the potential effects of these environmental conditions in the growth of replicators in the reactor. 

The mathematical description in Equations (1) and (2) also resembles the definitions of vesicle-like compartments or “protocells” [18,31,32], in which the replicators undergo an autocatalytic process in a confined volume. Although both are open systems (*i.e.*, considering inlets *and* outlet streams of monomers) coupled to the surroundings, the model here is macroscopic, so stochastic effects due to low replicator concentrations are neglected [18]. 

### 2.2. A Mathematical Condition for Sustained Diversity

As mentioned in the introduction, the definition of coexistence posed here is that multiple autocatalytic replicators sustain linear growth rates simultaneously, despite sharing common resources. Here, a mathematical description of this coexistence scenario is provided, based on the kinetic, composition and environmental parameters in Equations (1) and (2). In the particular case of the chemical system described by Equation (1), with *n* = 2 replicators, *x*_1_, *x*_2_ and λ = 2, and monomers, *m_a_*, *m_b_*, the replicator growth rate is written as:

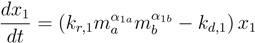
(3)

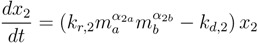
(4)


According to the proposed linear behavior, one may express the replicator concentration profiles as *x*_1_ = *c*_1_*t* and *x*_2_ = *c*_2_*t*, where *c*_1_ and *c*_2_ represent the constant growth rates of each replicator. The value of the initial concentration of the replicators inside the system (which would correspond to the intercept of these lines) is neglected, relative to the long-term replicator populations. Using these proposed linear replicator profiles, and noticing that $\frac{dx_{1}}{dt}=c_{1}$, $\frac{dx_{2}}{dt}=c_{2}$, Equations (3) and (4) may be rearranged to solve for the monomer concentrations:

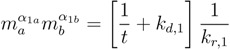
(5)

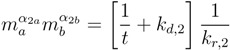
(6)


Since the idea is to evaluate the sustained diversity of the replicators over long periods of time, one can assume that $t\gg \frac{1}{k_{d,i}}$. Using this assumption, Equations (5) and (6) become a nonlinear system of equations for the monomer concentrations, *m_a_* and *m_b_*, that enables the simultaneous linear growth of both replicators when *c*_1_ and *c*_2_ are positive. Notice then that the values of the monomer concentrations depend on the kinetic parameters of the replicators, but not on the composition of either replicator or on the environmental parameters. The solution of the nonlinear system of equations in Equations (5) and (6) is:

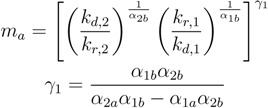
(7)

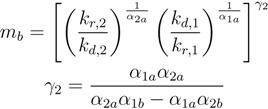
(8)


These long-term monomer concentrations do not depend on variables that change over time. Equations (7) and (8) suggest that for a linear replicator growth, it is necessary that the monomer concentrations inside the fed-batch reactor reach a steady-state. The steady-state solution of the monomer concentration, *m_a_* and *m_b_*, can be derived by writing $\frac{dm_{j}}{dt}=0$ on the left-hand side of Equation (2). In addition, the right-hand side of this equation may be expressed in terms of the replicator growth rates, *c*_1_, *c*_2_, in order to construct a system of linear algebraic equations to solve for these unknown replicator growth rates. The steady-state equations for *m_a_* and *m_b_*, according to Equation (2), are:

$$\frac{Fm_{in,a}}{V}-\frac{Fm_{a}}{V}=L_{1a}c_{1}+L_{2a}c_{2}$$ (9)

$$\frac{Fm_{in,b}}{V}-\frac{Fm_{b}}{V}=L_{1b}c_{1}+L_{2b}c_{2}$$ (10)

Equations (9) and (10) explain the rationale behind the assumption of a linear behavior for the replicators. At the beginning of the replication process, the replicators exhibit an exponential concentration profile, due to the initial concentration of monomers in the bioreactor and the addition of monomers in the system via the inlet flow $\frac{Fm_{in,j}}{V}$. This means that the replicator growth rates, *c*_1_, *c*_2_, are positive and increase rapidly. During the simulation, the monomer concentration decreases, due to the consumption by the replicators and the external outlet flow, $\frac{Fm_{j}}{V}$, which allows some food resources to leave the system. At the same time, the replicator growth rates reach a maximum value and, then, slowly decrease their value, *but they continue to be positive*, which means replicators are continuously being formed inside the bioreactor at a lower rate. Finally, the material balance in Equation (2) starts to reach a steady-state value as the replicator growth rates become equal to the rate of food supply to the bioreactor in Equations (9) and (10). The value of this steady-state for the monomer concentration is provided in Equations (7) and (8). In other words, the monomers *A* and *B* are the limiting reactants of the reactions, and the replication process cannot grow faster than the constant monomer supply rate. 

Given the kinetic parameters of the autocatalytic replicators and the composition parameters, *L*_1_*_a_*, *L*_1_*_b_*, *L*_2_*_a_*, *L*_2_*_b_*, one may evaluate if the two replicators will exhibit the coexistence linear behavior. If the replicator growth rates, *c*_1_ and *c*_2_, in Equations (9) and (10) are both positive, sustained coexistence may be possible, according to the linear growth definition proposed here. Equations (9) and (10) also incorporate elements from the environment via the inlet flow rate, *F* , and monomer concentrations, *m_in,a_*, *m_in,b_*, highlighting the importance of including the environment in the modeling of coexistence. 

## 3. Analysis

Coexistence of distinct species provides a mechanism to increase chemical diversity on the prebiotic Earth, allowing new species generated through mutation or spontaneous generation to take hold in the population. The model is used here to identify parameter sets that exhibit coexistence. Once the parameter sets are identified, suitable chemistries and environments can then be selected or designed. 

The analysis is performed for a simple, but illustrative, case, with *n* = 2 replicator types and *λ* = 2 monomer types. Each replicator type is formed by 20 monomers. Given these four different chemical species, the parameter space of the model contains eight kinetic parameters (*k_r_*_,__1_, *k_r_*_,__2_, *k_d_*_,__1_, *k_d_*_,__2_, *α*_1_*_a_*, *α*_1_*_b_*, *α*_2_*_a_*, *α*_2_*_b_*) and two composition parameters (*L*_1_*_a_*, *L*_2_*_a_*). The exploration of the parameter space is made in two parts; first, a random sample selection is generated, having 10,000 different kinetic parameter sets using a statistical Latin Hypercube approach [33] with the following upper and lower bounds.

1 × 10^−^^4 ^≤ *k_r,i_*, *k_d,i_* ≤ 1 × 10^3^   *i* = 1, ..., *n* (11)

0.1 ≤ *α_j,i_* ≤ 3   *i* = 1, ..., *n*   *j* = 1, ..., λ (12)

Then, for each of the 10,000 kinetic parameter sets that have been generated, the ordinary differential equations (ODE) in Equations (1) and (2) are simulated using multiple combinations of the composition parameters, *L_ij_*. The equations are solved in MATLAB v.R2012a, using solver *ode15s*, due to the several orders of magnitude in the evaluated replication and dissociation rate constants that causes stiffness in the ODEs. The evaluation of *L_ij_* is constrained by forcing both replicator types to have at least one building unit of each monomer type. Therefore, since both replicators have a length of 20 units, 361 (19 × 19) possible combinations of the composition parameters are evaluated for each kinetic parameter set. Table 1 presents a summary of all nominal conditions for each of these simulations. 

**Table 1:** Summary of nominal values for the mathematical model in Equations (1) and (2), *n* = 2, λ = 2.

Variable	Description	Units	Value
*x*_0__,__1_	Initial concentration, *x*_1_	mol/m^3^	0.1
*x*_0__,__2_	Initial concentration, *x*_2_	mol/m^3^	0.1
*m*_0__,*a*_	Initial concentration, *m_a_*	mol/m^3 ^	0.5
*m*_0__,*b*_	Initial concentration, *m_b_*	mol/m^3^	0.5
*m_in,a_*	Inlet concentration, *m_a_*	mol/m^3^	0.5
*m_in,b_*	Inlet concentration, *m_b_*	mol/m^3^	0.5
*F*	Inlet flow rate	m^3^/s	1
*V*	Reactor volume	m^3^	1
*t_f_*	Simulation time	s	1 × 10^7^
*L_ia_* + *L_ib_*	Total replicator size	monomers	20

Notice in Table 1 that the simulation time, (*t_f_* = 1 × 10^7 ^s), is several orders of magnitude greater than the decomposition timescales, $\left(\frac{1}{k_{d,i}}\right)$, in order to ensure the resolution of the system dynamics and the evaluation of the sustained diversity of the replicators over long periods of time. Although a wide range of parameters is included here, one should also note that other parameter sets not satisfying these constraints may also exhibit coexistence, according to the conditions specified in Equations (9) and (10). 

## 4. Results

### 4.1. Growth of Replicators under a Constant Monomer Supply

**Figure 2:**
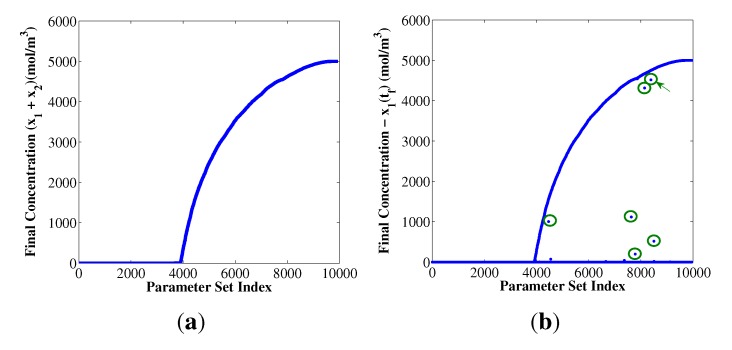
Exploration of kinetic parameter space for the replication process in a prebiotic environment. The parameter set index represents each of the 10,000 kinetic parameter sets used in this analysis, in ascendent order, according to the total concentration of replicators, (*x*_1_ + *x*_2_), at *t* =1 × 10^5 ^ s. The kinetic parameter sets inside the green circles indicate cases where both replicators are able to grow significantly relative to the initial conditions.

To organize the results of the large number of simulations in Section 3 (over 3.5 × 10^6 ^ODE simulations), the final total concentration of replicators, (*x*_1_ + *x*_2_ at *t* = *t_f_*), in each simulation is computed. For each of the kinetic parameter sets, only the combination of composition parameters that generates the maximum value of *x*_1_ + *x*_2_ at *t *= *t_f_* is selected, and then, the kinetic parameter sets are placed in ascendent order according to this value. Figure 2 summarizes the results of this ranked performance of the kinetic parameter sets. In Figure 2a, 38% of the kinetic parameters drive the system to have a final total replicator concentration lower than the initial total replicator concentration, (*x*_0__,__1_ + *x*_0__,__2_ = 0.2 mol/m^3^). Over a longer simulation time, *t_f_*, these parameters lead to the extinction of the replicators and any information that they may contain. 

As for the remaining 62% of the kinetic parameter space, there are two possible scenarios—either one of the replicator types is able to take over the system as a “single-winner” or both replicator species are able to coexist over the simulation time. Figure 2b illustrates this aspect by showing the final concentration of replicator, *x*_1_. It is clear from this figure that the most likely result in a constant monomer supply is a single-winner scenario (≃62% of the cases), since high *x*_1_ final concentration matches with the final total replicator concentration in Figure 1a, and low *x*_1_ final concentration indicates that the other replicator has taken over the system. 

**Figure 3:**
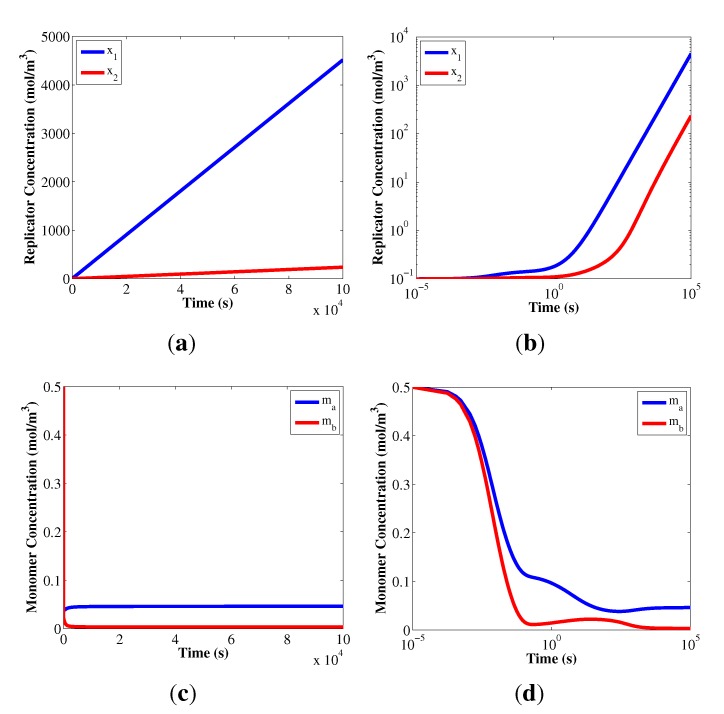
Example of replicator and monomer concentration profiles. These figures correspond to the parameter set indicated with a green arrow in Figure 2b. Table 1 indicates the simulation conditions for these figures; Table 2 shows the values of the kinetic parameter set used in the solution of Equations (1) and (2), *L*_1_*_a_*= 10, and *L*_2__a_ = 1. Figure 3b,d show the replicator and monomer concentration profiles on a logarithmic scale to highlight the initial transient response.

The main focus of this work is on the small fraction of the kinetic parameter space where both replicators are able to grow, despite sharing the monomers in the system. Among the parameter sets that exhibit coexistence at the end of the simulation, one of them is highlighted in Figure 2b with a green arrow, because it shows unique features for the sustained coexistence of both replicators, as shown in Figure 3. This figure indicates that the replicator concentration profiles are linear, in agreement with our definition of coexistence. These linear concentration profiles imply that this parameter set exhibits a balance between the kinetics and composition of the replicators, such that the overall replicator growth rate is equal to the constant rate at which the monomers are entering into the system. As a result of this net material balance, the replicator growth rates are sustained in the linear portion of the growth curve, and the monomer concentration levels off inside the system, reaching a steady state condition for both monomers, as shown in Figure 3c. 

Table 2 shows the values of the kinetic parameters that generate the linear behavior in Figure 3. Notice that the ratio between the replication and dissociation constants of the replicators is greater than one and similar in magnitude, 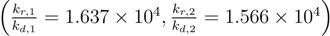. Therefore, the linear replicator profiles are based on how the reaction orders, *α_ij_*, and the composition parameters, *L_ij_*, are used to manage the shared monomer resources. 

Finding the right balance between αij and *L_ij_* is vital to developing the linear behavior observed in Figure 3a. For example, a simulation of the chemical system with the values in Table 2 and the parameters, *L*_1__a_ = *L*_2__a_ = 10, describes a more competitive scenario between the replicators, since both of them equally require both monomers. In this case, the result of the simulation is a *x*_2_ single-winner scenario. Notice both replicators are less sensitive to the B concentration, given the low reaction orders, *α*_1_*_b_* and *α*_2_*_b_*. To highlight the role of the reaction orders as a metric of the sensitivity of the replicators to the monomer concentrations, an additional simulation of the parameter set in Table 2 using *L*_1_*_a_* = 10, *L*_2_*_a_* = 1 and *α*_1_*_b_* = 0.6 was performed, resulting in a *x*_2_ single-winner scenario. These examples suggest that both replicators are in a constant competition for the shared monomers and that replicators with low reaction orders are more robust to monomer variations and, therefore, less coupled to the dynamics of the remaining replicators in the system.

**Table 2:** Kinetic parameter set that corresponds to the observed linear replicator profiles in Figure 3a,c using the nominal values in Table 1.

*k_r_*_,__1_ = 388.052	*k_r_*_,__2_ = 54.821
*k_d_*_,__1_ = 0.0237	*k_d_*_,__2_ = 0.0035
*α*_1_*_a_* = 2.4544	*α*_2_*_a_* = 2.0325
*α*_1_*_b_* = 0.3773	*α*_2_*_b_* = 0.5965

### 4.2. Condition of Coexistence for Autocatalytic Replicators

Section 2.2 describes how the system of linear equations in Equations (9) and (10) can be used to explain the observed linear behavior between the replicators for sustained diversity. If the solutions of this system of linear equations for the replicator growth rates, *c*_1_ and *c*2, are positive, then the desired linear behavior is possible. Here, Equations (9) and (10) are evaluated not only to screen for sustained diversity behavior, but also to screen for the complete extinction scenario. 

Table 3 summarizes the comparison between the results from Equations (9) and (10), with the observed results in each of the 3.61 × 10^6 ^different ODE simulations, evaluating all possible replicator compositions in the case study (*i.e.*, 361 possibilities) for each of the 10,000 parameter sets used to explore the kinetics of these replicators. Notice that the sum of the percentages in the total column adds to 92.45%. In addition, 2.19% corresponds to unsuccessful parameter combinations, whose results presented numerical instabilities during the ODE simulation. The remaining difference (5.36%) corresponds to the successful simulations where the composition of the replicators was the same, (*L*_1_*_a_* = *L*_2_*_a_*). These results are not included in Table 3, since these combinations do not provide a unique solution for the system of linear equations in Equations (9) and (10). Nonetheless, the cases where (*L*_1_*_a_* = *L*_2_*_a_*) will be discussed later in this section. 

**Table 3:** Results for the final scenario for two autocatalytic replicators following the mathematical model in Equations (1) and (2). The observed states correspond to the possible end results in the simulation: extinction of replicators, single-winner scenario (either *x*_1_ or *x*_2_) and coexistence of replicators. The screening states describe the possibilities for the solutions of the system of linear equations in Equations (9) and (10). The percentages were calculated over the results from 3.61 × 10^6 ^ODE simulations of different kinetic parameter sets and several replicator compositions, using the nominal values in Table 1.

Observed	Screening			Total
	*c*_1_ < 0 *c*_2_ < 0	*c*_1_ > 0, *c*_2_ < 0 *c*_1_ < 0, *c*_2_ > 0	*c*_1_ > 0 *c*_2_ > 0	
**Extinction**	1.87%	34.89%	0.00%	36.76%
**Single Winner**	0.00%	53.96%	0.83%	54.79%
**Coexistence**	0.00%	0.00%	0.90%	0.90%

The diagonal values in Table 3 indicate the percentages of successful identification of the corresponding observed final states. Notice that the system of algebraic equations provides a 100% accuracy in the identification of scenarios where both replicators are extinct, given the fact that the off-diagonal elements in the first column are all zeros. This result indicates that the system of algebraic equations does not generate a Type I error (or false positives) with its identification. 

The screening of the parameter space made by the system of algebraic equations in Equations (9) and (10) shows significant results for the case of the coexistence scenario. Table 3 indicates that coexistence between the replicators is only possible when *c*_1_ and *c*_2_ are both positive values. As for the quality in the identification of coexistence made by Equations (9) and (10), the probability of a Type I error for a particular parameter set is about 48%. These results indicate a key feature for the system of algebraic equations, since 0.9% of the entire parameter space leads to the coexistence of the replicators, and the system of algebraic equations is excluding most of the possible parameter combinations that can be evaluated for coexistence. 

Table 4 compiles the results from the ODE simulations in the parameter set exploration, when both replicators have identical composition (*i.e.*, *L*_1_*_a_* = *L*_2_*_a_*). This table indicates that there is not a set of kinetic and composition parameters that led to the sustained linear behavior. Therefore, the system of algebraic equations accounts for all possible parameter combinations that exhibit coexistence. Overall, satisfying the system of linear equations in Equations (9) and (10) is a necessary, but not sufficient, mathematical condition for the coexistence of autocatalytic replicators in the model. 

**Table 4:** Summary of the final results for two autocatalytic replicators with identical composition (*L*_1_*_a_* = *L*_2_*_a_*) following the mathematical model in Equations (1) and (2). The observed states correspond to the end results in the simulation: extinction of replicators, survival of a replicator (only *x*_1_ or only *x*_2_), and coexistence of replicators. The percentages were calculated over the results from 3.61 × 10^6 ^ODE simulations of different kinetic parameter sets and replicator compositions, using the nominal values in Table 1.

Extinction	Single Winner	Coexistence	Total
2.18%	3.19%	0.00	5.27%

**Figure 4:**
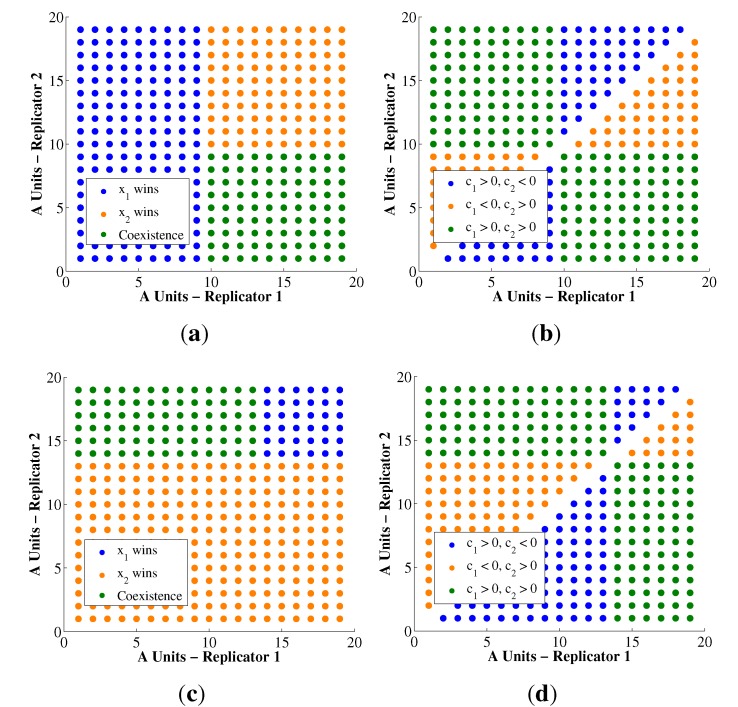
Results from the system of algebraic equations, Equations (9) and (10), for two different kinetic parameter sets. Figure 4a,b correspond to the parameter set in Table 2, Figure 4c,d correspond to the parameter set in Table 5. Figure 4a,c indicate with colors the final result in the simulations of these kinetic parameters for each possible combination of composition parameters. Figure 4b,d indicate the predicted replicator growth rates, *c*_1_ and *c*_2_, from the system of algebraic equations at each replicator composition. All simulations used the nominal values in Table 1.

Table 4 also suggests that it is necessary to have different replicator compositions in order to have coexistence. When both replicators have the same composition, the competition for monomer resources increases inside the system. In the case of *L*_1_*_a_* = *L*_2_*_a_*, coexistence is never observed, but rather, a single winner or extinction outcome. Having diverse replicator composition is a way to mitigate the kinetic competition between the replicators, as an additional degree of freedom in the coexistence of the system. Figure 4 shows the replicator composition map of the final result of the simulations and the predicted replicator growth rates for two particular kinetic parameter sets (these parameter sets are indicated in Table 2 and Table 5). 

Figure 4a,c present the final results obtained from the simulations. Most of the replicator composition cases where coexistence is present are located in the upper-left or the lower-right corners of the replicator composition space. This indicates that the two replicators minimize the competition for the shared resources in the system, by each of them emphasizing a different monomer type for its replication. For Figure 4a, *x*_1 _requires more monomer *A* and *x*_2_ requires more monomer *B*. The opposite scenario occurs for the Figure 4c. In addition, the dependency/coupling of a replicator with the dynamics of a particular monomer type also depends on the magnitude of the reaction orders in the kinetic law. Notice in Table 2 that *α*_1_*_a_* > *α*_2_*_a_* reinforces the *x*_1_ dependency on *A*, and *α*_2_*_b_* > *α*_1_*_b_* reinforces the *x*_2_ dependency on *B* (the opposite happens with the parameter values in Table 5). In this model, replicators cannot be completely decoupled from the monomer dynamics, but weakening this coupling provides a significant advantage for the system to reach the coexistence state. When the reaction orders are low, the replicators become more robust to variations in monomer concentration, which confers an advantage in a highly competitive scenario with low monomer concentrations. 

**Table 5:** Kinetic parameter set values used in Figure 4 as a comparison for the solutions of the system of linear equations, Equations (9) and (10).

*k_r_*_,__1_ = 4.3692	*k_r_*_,__2_ = 1.6605
*k_d_*_,__1_ = 4.16 × 10^−^^4^	*k_d_*_,__2_ = 4.83 × 10^−^^4^
*α*_1_*_a_* = 0.2914	*α*_2_*_a_* = 0.3585
*α*_1_*_b_* = 2.6602	*α*_2_*_b_* = 0.7387

Figure 4b,d show the prediction made by the system of algebraic Equations in (9) and (10) for two kinetic parameter sets, across the replicator composition space. The empty spots in the diagonal of these figures correspond to the cases of equal replicator composition, where the equations cannot generate a prediction. It is clear from these figures that the system of algebraic equations predicts two potential regions for coexistence of the replicators, in agreement with the ≈50% prediction result in Table 3.

The system of algebraic equations accurately predicts the coexistence in the area where both the reaction orders and the composition parameters are reinforcing the decoupling between the replicators, as it was mentioned previously. These results do not imply that the prediction of coexistence using Equations (9) and (10) depends on the reaction orders and composition parameters only. Equations (9) and (10) show that coexistence of autocatalytic replicators requires a balance among all the parameters in the model, not only αij and Lij. However, the specialization of the replicators towards one particular monomer type is a conceptual explanation for the observations in Table 2 and Table 5, as well as Figure 4, to determine which of the two quadrants will be the one to exhibit coexistence. 

Finally, the conjectures regarding the relationship between kinetics and composition of the replicators are limited to the scenario where the monomer inlet concentrations, *m_in,a_*, *m_in,b_*, and the lengths of both replicators *are equal *during the simulation. The system of algebraic equations in Equations (9) and (10) includes these variables into the parameter set to compute the coexistence condition. In the search for other coexistence scenarios, Equations (9) and (10) provide the relationship between the numerous degrees of freedom (kinetic, composition and environment) in this design problem. 

## 5. Discussion

Diversity is a prerequisite for selection and evolution, which motivates this study on chemical coexistence of replicating species. The kinetic model presented here includes a unique combination of features: monomer recycling, inlet and outlet monomer flow and variable reaction orders on the monomer concentration. Reversible linkages are a possible route to non-enzymatic replication in the prebiotic Earth, and this motivated the inclusion of recycling into the model. The monomer species may have been scarce in the prebiotic Earth, such that sustained replicator growth would be limited by the inlet flow rate of monomers into the system. Additionally, monomers may be lost in the outlet flow, so that a system that efficiently incorporates monomer would maximize its overall population. Monomer scavenged by one replicator type may be later transferred to another type via the recycling dynamic. Thus, aspects of cooperation, as well as competition are embodied in this model. The definition of coexistence presented here, that of sustained linear growth of multiple species, is in contrast to many common definitions of coexistence based on steady-state or oscillatory populations. This new definition is motivated by the model, in which the continuous inflow monomers, coupled with the monomer recycling, can yield a new dynamic behavior with sustained open-ended growth. 

In the simulation portion of this study, constraints were placed on the kinetic parameters, and constant values were used for the environmental parameters and initial conditions. However, the analysis in Section 2.2 yields an algebraic condition between the kinetics, composition and environmental parameters that is independent of the constraints used in the simulation study. In fact, it is possible to compute the steady-state monomer concentrations for any set of kinetic parameters, using Equations (7) and (8). Thus, any set of replicator chemistries consistent with Equation (1) could potentially exhibit coexistence. It is the relationship of the kinetic parameters (via the long-term monomer concentrations) to the composition and environment, that determines if coexistence is possible. As a result, any reversible linkage chemistry exhibiting replication could be a candidate for coexistence. In the simulation shown, the concentrations of *A* and *B* in the inlet stream were equal, and thus. coexistence was only possible when there was a dominant pairing of replicator and monomer (*i.e.*, 1 − *A* and 2 − *B* or 1 − *B* and 2 − *A*). However, with unequal inlet concentrations, other combinations of kinetic parameters might also exhibit coexistence.

The algebraic conditions for coexistence do not include the initial conditions of the replicators or the monomers, and sensitivity of coexistence to the initial conditions was not presented in this paper. However, simulations from a range of replicator and monomer initial conditions showed no sensitivity of coexistence behavior to the initial conditions of the system. This suggests that the stability of the coexistence solution is independent of the initial condition. Observation of the system defined by Table 2 provides a rationalization of coexistence stability based on the relationship between the reaction order and the composition. A regulating negative feedback is required to stabilize the coexistence, by aligning the dominant reaction orders with the compositions of the replicators. This argument does not involve initial conditions and suggests that the basin of attraction of the coexistence solution is either the entire space or, else, is the empty set. 

This study focused on a simple yet dynamically rich scenario with two replicator types and two monomer types. However, a similar analysis to that presented in Section 2 can be performed for other cases. For the case with two replicators, but one monomer type, competition dominates the dynamic, such that only a vanishingly small set of kinetic parameters could exhibit coexistence. This is analogous to the model of Pross [3], in which coexistence is only possible when the ratio of rate constants is identical for each replicator. In practice, this would be extremely unlikely. 

The dynamic model presented in this paper does not specify any explicit function, other than replication, and represents a very early stage of evolution. With a sustained pool of replicators, mutations would invariably also occur, enabling additional species to be generated. Depending upon their kinetics and composition, relative to the environment, these mutants might also be sustained within the system, further expanding its diversity. The definition of coexistence based on linear growth does not imply that the population sizes of the species are similar, only that all are growing. A species with a small population size could be viewed as insignificant, if population size is the only criterion. However, if that species has or develops a necessary function, it could be critical to the future performance of the system, even if its population is not large. Unlike other replicator studies, the model presented here does not aim to quantify replicator strength, via population size or other metrics, but rather focuses on the system-level dynamics. A diverse system could later develop complex function through cooperation, such as catalysis via a hypercycle [1], but this is only possible under coexistence of multiple species. The system could be the unit upon which selection would act, and a collection of such units could undergo Darwinian evolution, to develop new functions and evolve according to its environment. 

## 6. Conclusions

Coexistence of diverse replicating species is necessary for evolution, but many replicator models predict the emergence of a single winner. Here, we present a chemically plausible kinetics model with a unique combination of effects that exhibits coexistence of two species for approximately 1% of the parameter space. The replicating species are not directly interacting, but rather, are coupled through shared monomer resources. The model is specified by parameters for the kinetics, composition and environment. The key features of the model are variable monomer reaction order, inlet and outlet flow of monomers and monomer recycling. Under appropriate parameter values, the populations of both replicating species grow with a linear profile. Algebraic conditions are derived to predict which parameter sets can exhibit coexistence, and in the simulation study, over half of the parameter sets meeting this condition do, in fact, have the stable linear growth solution, indicating sustained coexistence. Observation of these simulations suggests that the stability of this linear growth solution requires a weakened coupling between replicators, in which each replicator type is dominantly paired with one distinct monomer type. The algebraic coexistence conditions indicate that any system of replicators consistent with the kinetic model could exhibit coexistence—under an appropriate combination of compositional and environmental parameters. In a diverse prebiotic soup of monomers and replicators, coexisting replicators could be selected by the environment. 
